# Colovesical Fistula Complicating the First Symptomatic Episode of Acute Diverticulitis in a Young Adult

**DOI:** 10.7759/cureus.35082

**Published:** 2023-02-16

**Authors:** Rabira R Dufera, Oluwaremilekun Tolu-Akinnawo, Benedict J Maliakkal

**Affiliations:** 1 Internal Medicine, Meharry Medical College, Nashville, USA; 2 Gastroenterology and Hepatology, Nashville General Hospital, Nashville, USA

**Keywords:** acute pyelonephritis, colon, diverticulosis, colovesical fistula, acute diverticulitis

## Abstract

Colovesical fistula is one of the known complications of acute diverticulitis. However, it is uncommon for a patient to present with a colovesical fistula without prior episodes of diverticulitis. In this case, we report a patient with acute diverticulitis presenting with a colovesical fistula with no antecedent history of any medical condition. The patient was treated with intravenous antibiotics and subsequently had a temporary laparoscopic colostomy.

Although colovesical fistula caused by diverticular disease was once considered a relative contraindication to laparoscopic resection, this method is now being increasingly employed by experienced surgeons. Compared with laparoscopic colon resection surgery for uncomplicated diverticulitis, the minimally invasive treatment of colovesical fistula requires a longer operative time and advanced surgical skills.

## Introduction

Diverticular disease is the most common etiology for the development of colovesical fistulas. Patients older than 60 years old have a 30% prevalence of diverticulosis, with up to 50% prevalence in those above 80 years of age [1]. However, patients younger than 40 years have a lower prevalence of less than 10%. About 15% of the patients with diverticulosis will develop diverticulitis at some point in their life [2]. The incidence of colovesical fistula in the presence of diverticular disease is 2% to 4%; however, the incidence can vary from 2% to 23% [2].

Acute diverticulitis with colovesical fistula should be suspected in patients presenting with lower abdominal pain with air in urine (pneumaturia) or feces in urine (fecaluria). In addition, patients can also present with symptoms of urinary tract infections such as urinary frequency, urgency, fever, or chills. More importantly, acute diverticulitis with colovesical fistula should be suspected in males who can be young with no underlying anatomical abnormalities or other underlying medical conditions who present with pyelonephritis or urosepsis. For diagnostic purposes, clinical presentation, imaging, including CT with oral or intravenous contrast or MRI, cystoscopy, and colonoscopy can be utilized [3]. We present an unusual case of acute diverticulitis presenting with pyelonephritis and colovesical fistula confirmed with CT of the abdomen and pelvis on both oral and intravenous contrast, which was managed with intravenous antibiotics and laparoscopic colostomy.

## Case presentation

A 36-year-old Hispanic male presented to the emergency department with lower abdominal pain for five days. The pain was initially confined to the lower abdomen but later involved both flanks. He described the pain as dull to cramp-like and reported varying severity. He reported no prior history of similar illness. He also did not have any prior history of urinary tract infection. In addition, the patient also complained of dysuria and frank blood in the urine. He noted episodic streams of air when voiding urine. He was febrile prior to admission, ranging from 102-104°F for two days, and took Tylenol intermittently.

**Figure 1:**
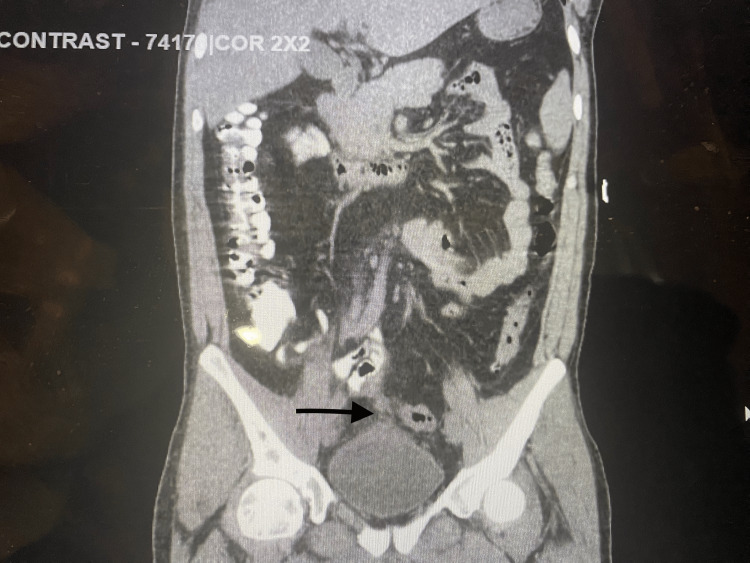
CT of the abdomen/pelvis with oral contrast. The black arrow indicates the colovesical fistula.

Notable vitals on arrival to the emergency were heart rate (HR) of 110 per minute, blood pressure (BP) of 108/88 mmHg, temperature of 102.3°F, and respiratory rate (RR) of 16 breaths per minute. On physical examination, the patient was found to have left lower quadrant abdominal tenderness, guarding, and rebound. Furthermore, labs on arrival showed leukocytosis of 16,000/mL with left shift (normal: 4,500-10,000/mL), serum sodium 131 mmol/L (normal: 136-145 mmol/L), serum potassium 4 mmol/L (normal: 3.5-5.1 mmol/L), blood urea nitrogen (BUN) 30 mg/dL (normal: 7-17 mg/dL), creatinine 1.45 mg/dL (unknown baseline, normal: 0.55-1.02 mg/dL), and lactic acid 2.2 mmol/L (normal: 0.4-1.9 mmol/L). The liver function test (LFT) was within normal limits. Urinalysis was positive for nitrites, leukocyte esterase, +1 bacteria, and +1 blood. CT of the abdomen and pelvis with oral contrast was done and confirmed colovesical fistula (Figure 1).

**Figure 2:**
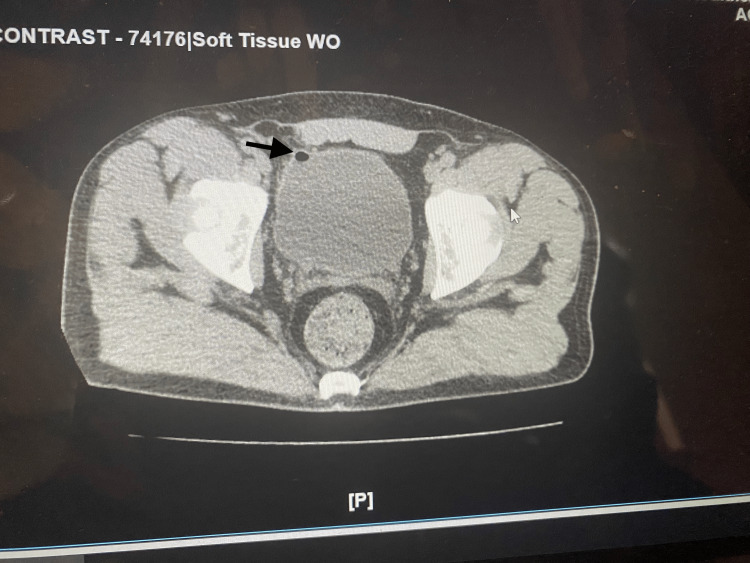
CT of abdomen/pelvis with intravenous contrast. The black arrow shows the colovesical fistula with air in the bladder.

CT of the abdomen/pelvis with intravenous contrast also showed perinephric inflammatory changes surrounding both kidneys with mild edema, which suggested underlying pyelonephritis, diverticulosis with diverticulitis of the sigmoid colon, and colovesical fistula with inflammatory thickening of the superior dome of the bladder secondary to adjacent diverticulitis (Figure 2).

The patient was started on 1 g of intravenous ceftriaxone over 24 hours and bowel rest. A nasogastric tube was placed for decompression pending further surgical intervention, as there was also a concern for bowel obstruction clinically. The patient continued further management with Tylenol, ceftriaxone, morphine, and Zofran. The intravenous antibiotic was later escalated to 4.5 g intravenous Zosyn every six hours. The patient’s blood culture remained negative; however, the urine culture grew extended-spectrum beta-lactamases (ESBLs) *Escherichia coli* (*E. coli*).

After initial stabilization, the patient underwent laparoscopic lysis of adhesions and placement of end colostomy. The procedure was performed by a general surgeon. She induced pneumoperitoneum by a Veress needle, and a 10-mm optical trocar was placed in the right para-umbilical region. Splenic flexure was mobilized with careful dissection. After identification and preservation of the left ureter under the Gerota fascia, she detached the strict adhesions between the sigmoid colon and the bladder. During these maneuvers, the attempt to identify the colovesical fistula was not successful due to extensive adhesion, and the decision was made to perform an end colostomy. There were also extensive adhesions of sigmoid to the surrounding omentum. Those adhesions were carefully released. Other intraoperative findings included sigmoid diverticulitis with a large inflammatory mass involving the rectosigmoid area. There was no resection of this inflammatory mass. The patient’s preoperative urinary bladder catheter was left in place and removed after seven days postoperatively. However, there was no resection of the urinary bladder. The patient’s postoperative course was uncomplicated. He was discharged home to follow up with general surgery for later colostomy reversal, takedown of colovesical fistula, and with a gastroenterology clinic appointment for colonoscopy in six to eight weeks. The patient was seen in the follow-up clinic and had no complications. He was able to resume normal activities within a few weeks.

## Discussion

Acute diverticulitis is common, but colovesical fistula from acute diverticulitis is a rare complication, occurring in less than 5% of cases. This case highlights the importance of suspecting colovesical fistula in patients with diverticulitis and urinary symptoms. Early diagnosis and prompt surgical intervention can prevent complications and improve patient outcomes, as in this case. Diverticular disease in Western countries usually involves the sigmoid colon. Fistulas most frequently also arise from this segment. The most common types of fistulas are colovesical (65%) and colovaginal fistulas (25%), followed by coloenteric (7%), colouterine (3%), oculocutaneous fistulas, and complex fistulas with involvement of multiple pelvic organs (10%) [2].

Incidence

While the precise incidence of colovesical fistula from diverticulitis remains unknown, there is a 2-3:1 male predominance [2]. This is attributed to the uterus protecting the bladder from inflamed sigmoid in females [2].

Etiology

The most common causes of colovesical fistula include diverticular disease (70%), inflammatory bowel disease including Crohn’s disease, malignancies, trauma, and iatrogenic causes [2,4,5].

Clinical features and diagnostic approaches

Patients with recurrent diverticulitis usually have low abdominal pain with a history of constipation. Typically, they are older patients in their 50s-60s. However, in atypical cases like our patient, patients may present with a short duration of abdominal pain. They may even present with symptoms of urinary tract infections such as dysuria, frequency, urgency, or, more importantly, may present with complicated pyelonephritis in relatively young patients in their 30s. Of note, patients with colovesical fistula present with pneumaturia and/or fecaluria. Pneumaturia is more common than fecaluria. Overall, 70-90% will have pneumaturia, whereas about 50-70% usually have fecaluria. Another 87.5% will present either with pneumaturia or fecaluria [2,4,5]. Other less frequent symptoms include chronic abdominal pain, diarrhea, hematuria, and loss of urine via the rectum [2,4,5].

CT of the abdomen with oral and intravenous contrast is the most frequently performed imaging modality. It is sensitive in detecting fistula in about 76% and diagnosing underlying etiology in about 94%. CT findings suggestive of acute diverticulitis include localized bowel wall thickening (>4 mm) and an increase in soft-tissue density within the peri-colonic fat secondary to inflammation. The CT findings suggestive of colovesical fistula include contrast extravasation to the urinary bladder or the presence of gas in the urinary bladder with no recent urinary bladder catheterization [2,4,5]. Colonoscopy is sensitive to detect underlying etiology such as a colonic malignancy in more than 95%. Cystoscopy is less sensitive in detecting colovesical fistula. It is mainly used if there is suspicion of bladder cancer-associated colovesical fistula. Cystoscopy is also recommended if there is no colonic pathology as the underlying cause of colovesical fistula. On cystoscopy, the fistula tract is rarely seen, but there may be a focal edematous area of the bladder wall. MRI can also assist in detecting complex fistulas [2,4,5].

The management of colovesical fistula is primarily by surgery. However, if the patient is not clinically stable due to untreated infection, the infection should be treated prior to fistula repair. Preoperatively, the patient should have urinary bladder catheterization for decompression. The bladder side of the fistulous tract often does not require any intervention beyond decompression with a Foley catheter. However, if the fistula opening is visible, it can be closed with simple closure. Urinary bladder catheter should remain for five to seven days postoperatively. For most benign colovesical fistulas, single-stage resection with primary anastomosis is acceptable; however, a multistage operation is necessary for patients at risk of anastomotic leak or for patients who cannot tolerate prolonged procedures. Patients with large abscesses, unstable patients, and patients on corticosteroids should be managed with multistage operations due to the risk of anastomotic leak. Laparoscopic resection of the colovesical fistula, on the other hand, can be performed for patients who developed a colovesical fistula due to diverticulitis in experienced hands. Early suspicion and management of acute diverticulitis and its complications in younger patients who are 50 years old or less are crucial as several studies have shown a more aggressive course in this age group [6-11]. They are more likely to have recurrent diverticulitis, which may predispose them to complications, including perforations and the formation of colovesical fistulas.

## Conclusions

This case highlights the importance of early diagnosis and prompt surgical intervention in the management of acute diverticulitis with colovesical fistula. It also emphasizes the need for a high clinical index of suspicion for colovesical fistula, particularly in patients with an atypical presentation, regardless of their age. In addition, it also highlights surgical interventions and follow-up afterward. Furthermore, there is a need for additional studies to identify those at increased risk of these complications so that appropriate preventative measures can be introduced early.
